# Government Economics of Expanding Canada’s Medical Assistance in Dying to Vulnerable Populations and the Ethical Implications of Allowing the State to Control Death

**DOI:** 10.1177/00302228251323299

**Published:** 2025-02-28

**Authors:** Uzair Jamil, Joshua M. Pearce

**Affiliations:** 1 6221 Western University, London, ON, Canada; 2 Ivey School of Business, 6221 Western University, London, ON, Canada

**Keywords:** euthanasia, medical assistance in dying, state killing, ethics, vulnerable populations, economics

## Abstract

This study explores the potential economic savings from expanding medical assistance in dying (MAiD) in Canada, where it is currently a leading cause of death, to include vulnerable groups that cost the government more than they contribute in taxes. These groups include individuals with severe mental health issues, the homeless, drug users, retired elderly, and indigenous communities. Both voluntary and non-voluntary scenarios were analyzed, projecting total savings of up to CAD $1.273 trillion by 2047. With an estimated 2.6 million deaths in the voluntary scenario, mostly among mentally ill and elderly populations, this cost-saving measure raises significant ethical concerns. Financially incentivizing MAiD could shift healthcare priorities away from providing necessary support, potentially devaluing vulnerable lives and fostering a troubling reliance on assisted death as an economic solution. The findings highlight a need for ethical scrutiny of MAiD policy expansion.

## Introduction

The Canadian government legalized euthanasia or medical assistance in dying (MAiD) for eligible citizens in 2016 (D. of J. Government of Canada, 2021). This policy change has resulted in a marked increase in the country’s mortality rate, with MAiD now constituting the fifth leading cause of death in Canada (Passifiume, 2024; W. J. Smith, 2024). MAiD was predominantly first used for patients with terminal illnesses experiencing intolerable suffering (Government of Canada, 2023b). In the last two years, however, this has been expanded to those that are not terminally ill (non-foreseeable deaths) at an accelerating rate (Government Of Canada, 2023a). Ongoing legislative proposals, seek to broaden eligibility further to include Canadians with severe mental health conditions (Dyer, 2024; D. of J. Government of Canada, 2016). For instance, Bill C-14 states that individuals with mental illness or physical disabilities will not be excluded from MAiD (D. of J. Government of Canada, 2016). Currently, Canadians seeking MAiD must voluntarily request it, be at least 18 years old, and possess the capacity to make informed healthcare decisions (Government of Canada, 2023). They must also provide informed consent and be eligible for publicly funded healthcare services in Canada (Government of Canada, 2023). Additionally, the person must be experiencing physical or mental suffering deemed unbearable, with no relief options considered acceptable (Government of Canada, 2023). Proponents of extending MAiD to mental illnesses argue that such conditions are as legitimate as physical illnesses in terms of suffering (Bahji & Delva, 2022; Dembo et al., 2018; Dying With Dignity Canada, 2024; Kamm, 1997; Martin, 2023; Stoll et al., 2021).

In 2022 there were 13,241 MAiD cases, accounting for 4.1% of all deaths in Canada (Government of Canada, 2023). The cumulative total of MAiD since the program’s start has reached 44,958 (Government of Canada, 2023). A 31.2% rise in MAiD cases was observed in 2022, which is around the 32.6% increase observed in the preceding year (Government of Canada, 2023). Projections for MAiD extended to mental illness suggest a significant increase with cases surging to between 227,024 to 896,312 cases by 2047 according to conservative estimates and on the high end up to approximately 4.9 million by 2047 (which would impact 12.3% of the population) (Jamil & Pearce, 2024).

Canada itself is in economic decline (Fraser Institute, 2024), which is stressing the medical system. This decline represents the second-longest and third-deepest drop in living standards during this period (Fraser Institute, 2024). Canada’s weak economic growth has persisted for a decade, with per capita real GDP showing its smallest increase in nearly a century (Cross, 2023). Canada’s quality of life index ranking has deteriorated from 5^th^ place in 2013 (Numbeo, 2013) to 30^th^ place in 2024 (Numbeo, 2024). A rapid increase in immigrant population has exacerbated pressures on affordable housing, schools and hospitals (Bochove et al., 2024; Silberman, 2024). Housing affordability reached its lowest point in over 40 years (Lord, 2023) and is linked to adverse effects on i) community health (Pollack et al., 2010), ii) crime rates (Gabriel & Painter, 2020), iii) homelessness (Moore & Skaburskis, 2004), iv) children’s wellbeing (Harkness & Newman, 2005) and v) economic growth (Du & Zheng, 2020). In 2022, 35% of Canadians reported difficulty meeting their financial needs over the past year, and 26% indicated they could not cover an unexpected expense of $500 (S. C. Government of Canada, 2023a). A 2023 report suggests that more than 50% of Canadians were within $200 of being unable to pay all their bills (CTV, 2023).

Similarly, overcrowding is making schools worse for students in Canada (Moman, 2024), with students sharing desks and learning in non-traditional spaces (Ferguson, 2023). Ontario, de-streamed (eliminated both gifted and special needs cohorts) in part to maximize class sizes to handle the burgeoning student population.

Of these population pressures, the most severe is in healthcare. Canada’s healthcare system is facing a severe crisis (Canadian Medical Association, 2022; Labrie & Barua, 2023). Patients experience some of the longest wait times for essential medical care among developed nations (Labrie & Barua, 2023). Canada is projected to face a shortage of 117,600 nurses by 2030 and 78,000 physicians by 2031 (House of Commons Canada, 2023). Canadians also experience one of the highest costs for a universal healthcare system (Labrie & Barua, 2023). Canada’s per capita health care spending was one of the highest globally (Canadian Institute for Health Information, 2022) and overall health expenditure is expected to reach $344 billion, or $8740 per person/year (Canadian Institute for Health Information, 2024). The situation is deteriorating, with wait times reaching an unprecedented 27.4 weeks in 2022 (Labrie & Barua, 2023). Due to the delays during 2022-23, over 17,000 patients died while waiting for surgery or diagnostic scans (Lucyk, 2023).

There are many approaches to cutting Canada’s health care costs and improving efficiency such as reducing redundancy and using open source software (Peplinski & Pearce, 2023), however, several analysts have proposed killing patients as a cost saving measure. Smith suggested that the legalization of physician-assisted suicide (PAS) not only alleviates the suffering of terminally ill individuals, but also conserves medical resources and federal funds (W. M. Smith, 2017). Shaw and Morton present three economic arguments in favor of allowing assisted dying, including the notion that “the resources consumed by patients who are denied assisted dying could instead be used to provide additional (positive) quality-adjusted life years for patients elsewhere in the healthcare system who wish to continue living and to improve their quality of life” (Shaw & Morton, 2020). Similarly, Riley and Sarbey suggest that one potential benefit of MAiD could be the reduction in overall healthcare costs, leading to improvements in the health system (Riley & Sarbey, 2022). Trachtenberg and Manns calculated that the introduction of MAiD could alleviate healthcare spending in Canada by between $34.7 million and $138.8 million, surpassing the direct costs of implementation estimated between $1.5 million and $14.8 million (Trachtenberg & Manns, 2017). In 2021, the cost savings from MAiD, considering administrative expenses, were estimated at $86.9 million based on 6465 MAiD-related deaths (Bernier et al., 2020). With Law C-7, which allows non-terminally ill patients to use MAiD, it could result in an additional 1164 MAiD-related deaths, yielding $62 million in additional cost savings and an overall savings of approximately $149 million (Bernier et al., 2020).

Following this thought process, MAiD could be expanded to other vulnerable population groups, such as attempted suicide patients, which represent a demographic where it could be predicted with a high degree of certainty that all of them would make the choice to end their lives. Similarly, citizens with severe mental health problems would likely opt in. The homeless and drug users also face unique challenges in terms of stability and substance dependency and may select ending their lives (Gomez et al., 2010). Similarly for the retired elderly, some may select MAiD, but non-voluntary euthanasia in some European cases exist (Bilsen et al., 2009; Heide et al., 2007) and in 2006, the Royal Dutch Medical Association had proposed that simply being over 70 and weary of life could be seen as a legitimate basis for requesting euthanasia (Sheldon, 2005). Finally, indigenous communities in Canda may also be viewed as good candidates as they are particularly marginalized (Allan & Smylie, 2015; Kirmayer et al., 2014; Smye et al., 2023), with high rates of substance abuse (Firestone et al., 2015), suicide (Kumar & Tjepkema, 2019), and poverty (Anderson & Collins, 2014). Historical practices, such as elders sacrificing themselves for the community (Spearim, 2020; Stegman, 2022), indicate a complex relationship with end-of-life decisions, yet current leaders voice strong opposition to expanded euthanasia (Agnieszka, 2021).

This study is exploratory in nature, aiming to provide initial insights into the potential social, ethical, and economic impacts of MAiD scenarios across different populations in Canada. This paper will quantify current economic savings and costs from present MAiD (voluntary patients unwilling to bear either physical or psychological hardship), then extrapolate MAiD cost savings to the most likely areas of cost saving measures for other demographic groups where cost to the Canadian government is more than the tax revenue generated by the group: i) attempted suicide patients, ii) citizens with severe mental health problems, iii) homeless, iv) drug users, v) retired elderly, and vi) indigenous communities. For each demographic group, the number of potential MAiD patients and economic savings for the Canadian government will be quantified based on voluntary and non-voluntary scenarios. It should be noted, that in the demographic group for attempted suicide and current MAiD projections, there is no differentiation between voluntary and involuntary preferences for MAiD. Next, the costs to expand MAiD to each demographic group will be calculated using current costs, as well as using government-certified professional MAiD non-medical practitioners (or outsourcing). Finally, the results will be discussed in terms of the ethical challenges to overcome these cost savings measures.

## Methods

The data for this study were obtained from a variety of credible sources, including government publications, peer-reviewed articles, and independent research reports. The current cost for MAiD procedures when compared to the healthcare savings from avoided medical expenses, is derived from “Cost Estimate for Bill C-7 “Medical Assistance in Dying”” (Office of the Parliamentary Budget Officer, 2020). Data on suicide prevalence and associated costs were obtained from the *Canadian Association for Suicide Prevention* (Canadian Association For Suicide Prevention, 2024) and the *Public Health Agency of Canada* (P. H. A. of Canada, 1998, 2022). Mental illness prevalence and cost impacts were informed by Statistics Canada’s studies on mental health (S. C. Government of Canada, 2018b; 2023b) and the RiskAnalytica report *The Life and Economic Impact of Major Mental Illnesses in Canada* (Smetanin et al., 12/11). Homelessness data, including prevalence and associated costs, were sourced from *Homelessness Statistics in Canada* (2024) (Blair, 2024), Eynan et al. (Eynan et al., 2002), and the Parliamentary Budget Office’s *Federal Spending to Address Homelessness* report (Segel-Brown, 2024). Information on the financial burden and medical expenses of the elderly population was gathered from Statistics Canada reports (S. C. Government of Canada, 2019, 2022) and the study by Chen et al. (Chen et al., 2023). Indigenous demographic data and costs were derived from Statistics Canada (S. C. Government of Canada, 2018a; Kumar & Tjepkema, 2019), Parrott and Filice (Parrott & Filice, 2023), and the Department of Finance’s *Chapter 6: A Fair Future for Indigenous Peoples* (D. of F. Canada, 2024, p. 6). The dataset underwent cross-validation by two independent researchers to ensure its reliability and accuracy.

### Conventional Per-Person Cost of Administering MAiD

The total cost of administering a MAiD procedure is estimated at CAD$2327.32 (C_MAiD_ = 2327.32). This calculation assumes that every MAiD case results in the completion of the procedure, with no natural deaths occurring among these patients prior to MAiD being carried out.

### Outsourcing Approach to MAiD

An alternative approach could involve hiring government-funded professional dying assistants (killers) to carry out the MAiD procedure inside or outside hospitals. The lowest-cost hitmen that could be out sourced (OS) can provide the same function for as little as 150 Euros (Rios, 2010), which translates to approximately CAD $227 (C_MAiD,OS_ = 227), based on an exchange rate of 1 Euro = 1.51 CAD (xe, 2024a).

### Population Estimates Eligible for MAiD

#### Attempted Suicides

Research indicates that approximately 3.1% of Canadians have attempted suicide (F_suicide_ = 0.031) at some point in their lives (P. H. A. of Canada, 2022; Canadian Association For Suicide Prevention, 2024). Assuming that all individuals who have attempted suicide would opt for MAiD, the estimated number of individuals in this group choosing MAiD can be calculated by:

(1) $$N_{suicide}=F_{suicide}\times Pop\;\left[\text{people}\;\text{in}\;\text{Canada}\right]$$

Where Pop represents the total population of Canada, F_*suicide*_ is the fraction of the population who have attempted suicide and N_*suicide*_ is the projected number of Canadians who have attempted suicide and may opt for MAiD. This group does not differentiate between voluntary and involuntary choices, so only one cost is calculated.

#### Mentally ill

In 2022, over 5 million Canadians were diagnosed with a mood, anxiety, or substance use disorder (S. C. Government of Canada, 2023b). With a total population of 39.28 million in 2022 (S. C. Government of Canada, 2018b), this represents approximately 12.7% of the population (F_pop,mental_ = 0.127) who would potentially be eligible for MAiD if the eligibility criteria were expanded to include mental illness as has been proposed. For a conservative estimate, this scenario considers MAiD eligibility for individuals with severe mental health conditions—such as major depressive episodes, bipolar disorder, generalized anxiety disorder, social phobia, and substance use disorders including alcohol and cannabis use disorders—accounting for 72.4% of this group (F_severe-mental_ = 0.724), without imposing additional restrictions.

(2) $$N_{mental,non-vol}=F_{pop,mental}\times F_{severe-mental}\times Pop\;\left[\text{people}\;\text{in}\;\text{Canada}\right]$$

In the voluntary scenario, it is assumed that 3.1% of Canadians, the same proportion who have attempted suicide at some point in their lives, would voluntarily choose MAiD (F_mental,vol_ = 3.1%) (P. H. A. of Canada, 2022; Canadian Association For Suicide Prevention, 2024).

(3) $$N_{mental,vol}=F_{mental,vol}\times F_{pop,mental}\times F_{severe-mental}\times Pop\left[\text{people}\;\text{in}\;\text{Canada}\right]$$

#### Homeless

The approximate number of homeless individuals in Canada ranges from 150,000 to 300,000, with the numbers showing an upward trend (Blair, 2024). For the purposes of this evaluation, an average estimate of 225,000 is used (N_homeless,non-vol_ = 225,000). In non-voluntary scenarios, it is assumed that all individuals from the specified groups would opt for MAiD. In voluntary scenarios, based on a study by Eynan et al. (2002) (Eynan et al., 2002), 34% of homeless individuals have attempted suicide, and this proportion is used to estimate those who would voluntarily choose MAiD (F_homeless,vol_ = 0.34).

(4) $$N_{homeless,vol}=F_{homeless,vol}\times N_{homeless,non-vol}\;\left[\text{people}\;\text{in}\;\text{Canada}\right]$$

#### Retired Elderly

Approximately 19% of the Canadian population is aged 65 years and older (S. C. Government of Canada, 2019). Of this group, about 14.3% are between the ages of 65 and 79 (F_elderly,65-79_ = 14.3%) while 4.5% are aged 80 and above (F_elderly,80+_ = 4.5%). The total population of elderly individuals can thus be estimated using the following equation:

(5) $$N_{elderly,non-vol,65-79}=F_{elderly,65-79}\times Pop\;\left[\text{people}\;\text{in}\;\text{Canada}\right]$$

(6) $$N_{elderly,non-vol,80+}=F_{elderly,80+}\times Pop\;\left[\text{people}\;\text{in}\;\text{Canada}\right]$$

As of 2021, 4.51% of individuals aged 65 and older were living in poverty (S. C. Government of Canada, 2022). To segregate the individuals who would voluntarily opt for MAiD, this fraction of the populations is used (F_elderly,vol_ = 0.0451).

(7) $$N_{elderly,vol,65-79}=F_{elderly,vol}\times N_{elderly,non-vol,65-79}\;\left[\text{people}\;\text{in}\;\text{Canada}\right]$$

(8) $$N_{elderly,vol,80+}=F_{elderly,vol}\times N_{elderly,non-vol,80+}\;\left[\text{people}\;\text{in}\;\text{Canada}\right]$$

#### Indigenous Population

According to the 2021 Census, individuals identifying as indigenous constitute 5% of Canada’s total population (Parrott & Filice, 2023). As of 2016, The Inuit population is estimated to be 65,025 individuals (N_Inuit_ = 65,025) (S. C. Government of Canada, 2018a). The number of Indigenous people can be calculated using the following equation:

(9) $$N_{indigenous-Inuit}=\left(F_{indigenous}\times Pop\right)-N_{Inuit}\;\left[\text{people}\;\text{in}\;\text{Canada}\right]$$

The suicide rate among First Nations was approximately three times higher than that of the non-Indigenous population based on data from 2011 to 2016, while the rate among Inuit was nine times higher (Kumar & Tjepkema, 2019). These proportions will be used to determine the fractions for voluntary case.

(10) $$N_{indigenous,vol}=3\times F_{suicide}\times N_{indigenous-Inuit}+9\times F_{suicide}\times N_{Inuit}\left[\text{people}\;\text{in}\;\text{Canada}\right]$$

In the non-voluntary scenario, given that the poverty rate for indigenous individuals was 11.8% as of 2020 (F_poverty,ind_ = 11.8%) (S. C. Government of Canada, 2022), it is estimated that this percentage of the total indigenous population may be affected by MAiD.

(11) $$N_{indigenous,non-vol}=F_{poverty,ind}\times \left(N_{indigenous-Inuit}+N_{Inuit}\right)\left[\text{people}\;in\;\text{Canada}\right]$$

#### People Opting MAiD in the Current Scenario

The people opting for MAiD in the current scenario is given by Jamil et al. (equation (12)):

(12) $$M\;\left(t_{2016}\right)=146.7t_{2016}^{2}+765.49t_{2016}+426.57\;\left[\text{people}\;\text{in}\;\text{Canada}\right]$$

where t_2016_ denotes the number of years since the initiation of MAiD access in Canada, with year 1 starting in 2016 and $M\;\left(t_{2016}\right)$ is the number of MAiD case as per current rates in Canada.

### Savings from Providing MAiD to Individuals Eligible for MAiD

#### Attempted Suicides

In 2018, the total direct cost associated with suicide attempts was CAD $554 million which will amount to CAD $745 million as of 2024 (C_suicide,tot-2008_ = CAD $745 million) (D of F Canada, 2024; P. H. A. of Canada, 1998). With a total Canadian population of 37.26 million in 2018 (Pop_2008_ = 37.26 million) (S. C. Government of Canada, 2018b), the cost per individual who attempts suicide, can be calculated using the following equation

(13) $$C_{suicide}=\frac{C_{suicide,tot-2008}}{F_{suicide}\times {Pop}_{2008}}\;\left[\text{CAD}\$/\text{person}\right]$$

(14) $$S_{suicide}=C_{suicide}\times N_{suicide}\;\left[\text{CAD}\$/\text{person}\right]$$

#### Mentally ill

In 2011, the study conservatively estimated that the cost of mental illness was $42.3 billion in direct costs (Smetanin et al., 12/11). Taking into account that a dollar in 2011 is equivalent to 1.34 CAD in 2024, the total cost is calculated to be C_mental,direct-tot_ = CAD $56.9billion (D of F Canada, 2024; Bank of Canada, 2024). Considering this was distributed upon the whole fraction of the population diagnosed with mental health issues, the following equation can be used to estimate the cost per individual incurred due to mental health issues or that can be saved by administering MAiD:

(15) $$C_{mental,direct}=\frac{C_{mental,direct-tot}}{F_{pop,severe-mental}\times {Pop}_{2011}}\;\left[\text{CAD}\$/\text{person}\right]$$

(16) $$S_{mental,non-vol}=C_{mental,direct}\times N_{mental,non-vol}\;\left[\text{CAD}\$/\text{person}\right]$$

(17) $$S_{mental,vol}=C_{mental,direct}\times N_{mental,vol}\;\left[\text{CAD}\$/\text{person}\right]$$

#### Homeless

The annual budget allocated for homelessness programs at Infrastructure Canada is CAD $561 million (C_homeless,total_ = CAD $561 million) (Segel-Brown, 2024) so

$$S_{homeless,total,non-vol}=\text{CAD}\;\$561\;\text{million}$$

(18) $$S_{homeless,total,vol}=F_{homeless,vol}\times S_{homeless,total,non-vol}\;\left[\text{CAD}\$\right]$$

#### Retired Elderly

In 2018, the average expenditure per person aged 65 years and older was USD $12,411 (CAD $16,456, using the conversion rate of 1 USD = 1.36 CAD (xe, 2024b)), according to the Medical Expenditure Panel Survey commissioned by the U.S. Department of Health & Human Services. (Chen et al., 2023), which is $C_{retired,elderly}$ [CAD$/person].

It is assumed the average of age of individuals between 65 and 79 years as 72. Considering the life expectancy in Canada to be 81.6 years, these individuals are expected to live for 9.6 more years (Remaining life: R_L_ = 9.6). For 80 years and older, the life expectancy is considered to be a single year. Hence, the equation to determine the savings is given by:

(19) $$S_{elderly,non-vol,65-79}=N_{elderly,non-vol,65-79}\times C_{retired,elderly}\times R_{L}\;\left[\text{CAD}\$\right]$$

(20) $$S_{elderly,non-vol,80+}=N_{elderly,non-vol,80+}\times C_{retired,elderly}\;\left[\text{CAD}\$\right]$$

(21) $$S_{elderly,non-vol}=S_{elderly,non-vol,80+}+S_{elderly,non-vol,65-79}\;\left[\text{CAD}\$\right]$$

And for voluntary scenario,

(22) $$S_{elderly,vol,65-79}=N_{elderly,vol,65-79}\times C_{retired,elderly}\times R_{L}\;\left[\text{CAD}\$\right]$$

(23) $$S_{elderly,vol,80+}=N_{elderly,non-vol,80+}\times C_{retired,elderly}\;\left[\text{CAD}\$\right]$$

(24) $$S_{elderly,vol}=S_{elderly,vol,80+}+S_{elderly,vol,65-79}\;\left[\text{CAD}\$\right]$$

#### Indigenous Population

The Government of Canada has forecasted to spend CAD$32 billion per year on indigenous people in Canada for year 2024-25 (D. of F. Canada, 2024, p. 6). The cost incurred by the Canadian Government on indigenous population per capita is given by:

(25) $$C_{indigenous}=\frac{32,000,000,000}{N_{indigenous}}\;\;\left[\text{CAD}\$\right]$$

The potential savings from MAiD can be calculated from the following equations:

(26) $$S_{indigenous,vol}=C_{indigenous}\times N_{indigenous,vol}\;\left[\text{CAD}\$\right]$$

(27) $$S_{indigenous,non-vol}=C_{indigenous}\times N_{indigenous,non-vol}\;\left[\text{CAD}\$\right]$$

It should be noted that this is a conservative estimate because it only considers the extra costs for indigenous citizens, not the costs for all other citizens that the indigenous citizens could use as well.

#### People Opting for MAiD in the Current Scenario

To estimate the cost savings from MAiD as per current projections, the same proportions used in the document “Cost estimate for Bill C-7 Medical assistance in dying” were applied (Office of the Parliamentary Budget Officer, 2020) and summarized in Table 1. These proportions assume that 14% of individuals will have their lives shortened by 2 weeks, 25% by one month, 45% by three months, 13% by six months, and 3% by one year.

**Table 1.** Cost Savings due to Application of MAiD.

Conditions	Savings ($CAD)	Percentage
Expected death within 2-weeks – Cancer (C_2-weeks,C_)	11,428	F_2-weeks_ = 14%
Expected death within 2-weeks – Non-cancer (C_2-weeks,NC_)	10,987	F_2-weeks_ = 14%
Expected death within 1-month – Cancer (C_1-month,C_)	18,872	F_1-month_ = 25%
Expected death within 1-month – Non-cancer (C_1-month,NC_)	16,303	F_1-month_ = 25%
Expected death within 3-month – Cancer (C_3-month,C_)	34,977	F_3-months_ = 45%,
Expected death within 3-month – Non-cancer (C_3-month,NC_)	28,077	F_3-months_ = 45%,
Expected death within 6-month – Cancer (C_6-month,C_)	49,184	F_6-months_ = 13%
Expected death within 6-month – Non-cancer (C_6-month,NC_)	39,689	F_6-months_ = 13%
Expected death within 1-year – Cancer (C_1-year,C_)	67,963	F_1-year_ = 3%
Expected death within 1-year – Non-cancer (C_1-year,NC_)	57,408	F_1-year_ = 3%

The costs associated with each scenario is also mentioned in the report (Office of the Parliamentary Budget Officer, 2020). In addition, the costs are segregated as patients with cancer and without cancer. Accordingly, the proportions of MAiD patients are divided into 63% for cancer patients and 37% for non-cancer patients (F_cancer_ = 63% and F_non-cancer_ = 37%).

The following equations estimate cost savings from MAiD at current rate projections:

Cancer patients:

(28) $$S_{MAiD,cancer}=F_{cancer}\times M\left(t_{2016}\right)\left[F_{2-weeks}\times \left(C_{2-weeks,C}\right)+F_{1-month}\times \left(C_{1-month,C}\right)+F_{3-months}\times \left(C_{3-months,C}\right)+F_{6-months}\times \left(C_{6-months,C}\right)+F_{1-year,}\times \left(C_{1-year,C}\right)\right]\;\left[\text{CAD}\$\right]$$

Non-cancer patients:

(29) $$S_{MAiD,non-cancer}=F_{non-cancer}\times M\left(t_{2016}\right)\left[F_{2-weeks}\times \left(C_{2-weeks,NC}\right)+F_{1-month}\times \left(C_{1-month,NC}\right)+F_{3-months}\times \left(C_{3-months,NC}\right)+F_{6-months}\times \left(C_{6-months,NC}\right)+F_{1-year,}\times \left(C_{1-year,NC}\right)\right]\;\left[\text{CAD}\$\right]$$

### Cost Benefit Analysis

The cost benefit analysis is performed considering the cost incurred for providing MAiD for each individual, number of individuals opting and subtracting the product from the expected savings.

#### Attempted Suicides

(30) (CB)suicide=Ssuicide−(Nsuicide×CMAiD) [CAD$]

#### Mentally ill

(31) $${\left(C/B\right)}_{mental,non-vol}=S_{mental,non-vol}-\left(N_{mental,non-vol}\times C_{MAiD}\right)\;\left[\text{CAD}\$\right]$$

(32) $${\left(C/B\right)}_{mental,vol}=S_{mental}-\left(N_{mental,vol}\times C_{MAiD}\right)\;\left[\text{CAD}\$\right]$$

#### Homeless

(33) $${\left(C/B\right)}_{homeless,non-vol}=S_{homeless,non-vol}-\left(N_{homeless,non-vol}\times C_{MAiD}\right)\left[CAD\$\right]$$

(34) $${\left(C/B\right)}_{homeless,vol}=S_{homeless,vol}-\left(N_{homeless,vol}\times C_{MAiD}\right)\left[CAD\$\right]$$

#### Retired Elderly

(35) $${\left(C/B\right)}_{elderly,non-vol}=S_{elderly,non-vol}-\left(N_{elderly,non-vol}\times C_{MAiD}\right)\left[CAD\$\right]$$

(36) $${\left(C/B\right)}_{elderly,vol}=S_{elderly,vol}-\left(N_{elderly,vol}\times C_{MAiD}\right)\left[CAD\$\right]$$

#### People Opting MAiD in the Current Scenario

(37) $${\left(C/B\right)}_{MAiD}={\left(S\right)}_{MAiD}-\left(M{\left(t\right)}_{2016}\times C_{MAiD}\right)\left[CAD\$\right]$$

#### Indigenous

(38) $${\left(C/B\right)}_{indigenous,vol}={\left(S\right)}_{indigenous,vol}-\left(N_{indigenous,vol}\times C_{MAiD}\right)\left[CAD\$\right]$$

(39) $${\left(C/B\right)}_{indigenous,non-vol}={\left(S\right)}_{indigenous,non-vol}-\left(N_{indigenous,non-vol}\times C_{MAiD}\right)\left[CAD\$\right]$$

For the outsourcing scenarios, the cost associated with C_MAiD_ is replaced by C_MAiD,OS_.

## Results

### Deaths

If vulnerable populations were permitted to opt for MAiD in Canada, as identified in this study, the potential number of deaths could significantly increase beyond current projections. Based on estimates, 1,588,979 individuals who have previously attempted suicide could choose MAiD, while 4,713,015 individuals diagnosed with severe mental illness may also opt for it. Additionally, 225,000 homeless individuals, a group facing extreme socio-economic challenges, could be eligible under expanded criteria. Among the retired elderly population, approximately 9,687,649 individuals may seek MAiD as an option to address age-related health and quality of life concerns. When combined with current projections of MAiD uptake, which already stand at 175,143 deaths, these figures suggest a substantial increase in the number of individuals who may choose this end-of-life option. Similarly, as per estimates from this study, 302,419 individuals from the indigenous community might be subject to MAiD. The total number of potential MAiD deaths, if vulnerable populations were allowed to opt for it, amounts to *16,692,205* by 2047. In the voluntary scenario for MAiD, for individuals who have attempted suicide, the estimated number of deaths due to MAiD is 1,588,979. Among those with mental illness, the projected number of deaths is 146,103, reflecting a sizable fraction of this population who might choose MAiD. These projections were created by forecasting future MAiD cases based on current rate and then fitting an equation to those forecasts (Jamil & Pearce, 2024). For the homeless population, the estimate is 76,500 deaths. The retired elderly show an estimated 436,913 deaths under MAiD. The existing MAiD projections for the general population show an additional 175,143 deaths by 2047 while for indigenous population, it is estimated to be 250,442 deaths. The voluntary scenario will result in a total of *2,674,080* Canadian deaths. The inclusion of vulnerable populations into MAiD eligibility highlights the potential impact on both the healthcare system and the overall mortality rate in Canada, underlining the importance of ethical considerations and policy planning for such an expansion.

### Cost Benefit Analysis

The cost-benefit analysis for expanding MAiD under both non-voluntary and voluntary scenarios reveals significant financial implications for the Canadian government (Figure 1, Table 2, Table 3, Table 4 and Table 5).

**Figure 1.**
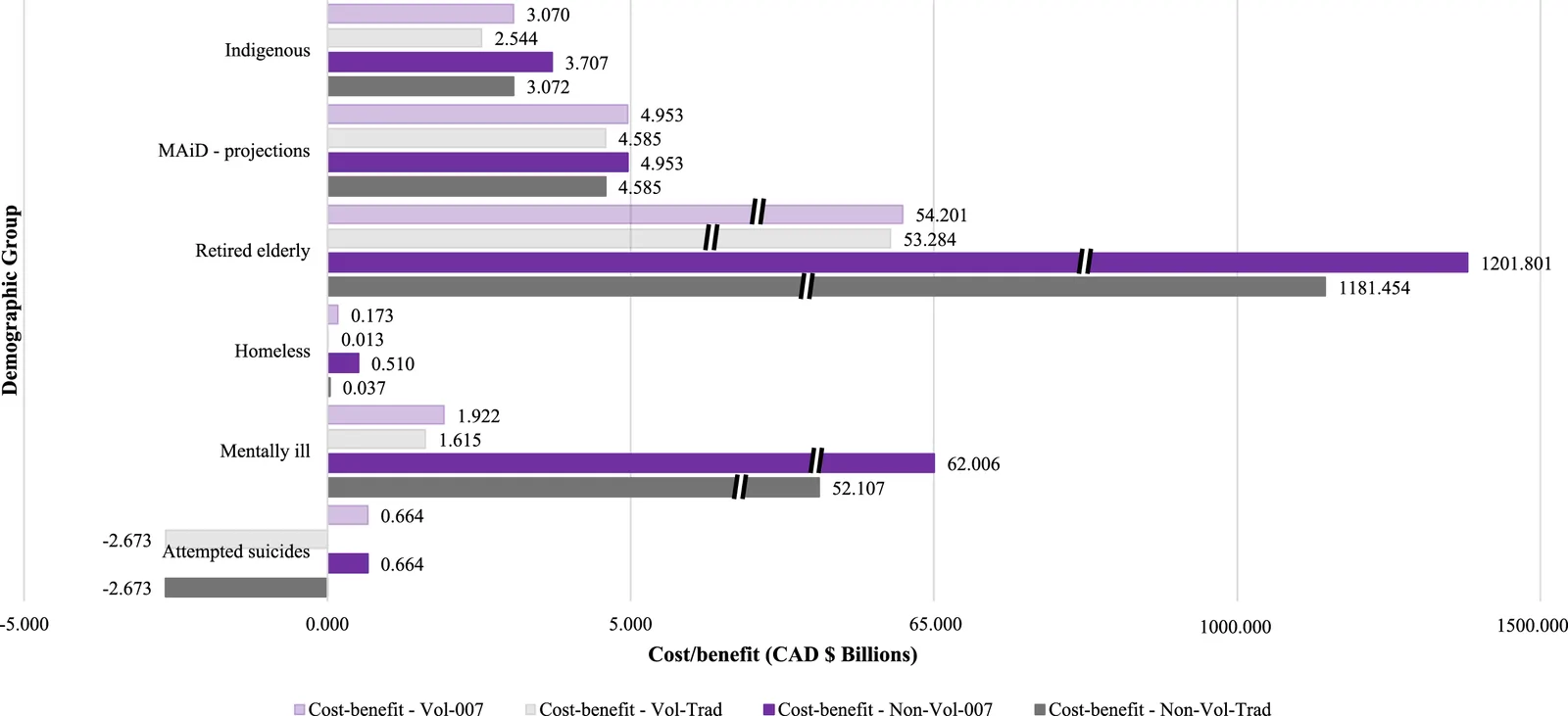
Cost/benefit analysis of performing MAiD with different approaches for both voluntary and non-voluntary scenarios.

**Table 2.** Non-voluntary Scenario With MAiD Performed Using Traditional Practice.

Scenarios	Costs ($ CAD billions)	Savings ($ CAD billions)	Cost-benefit ($ CAD billions)
Attempted suicides	3.698	1.025	−2.673
Mentally ill	10.969	63.076	52.11
Homeless	0.524	0.561	0.037
Retired elderly	22.546	1204.000	1181.454
MAiD - projections	0.408	4.993	4.585
Indigenous	0.704	3.776	3.072
Total non-voluntary traditional			1238.585

**Table 3.** Non-voluntary Scenario With MAiD Performed Using Outsourcing Approach.

Scenarios	Costs ($ CAD billions)	Savings ($ CAD billions)	Cost-benefit ($ CAD billions)
Attempted suicides	0.361	1.025	0.664
Mentally ill	1.070	63.076	62.006
Homeless	0.051	0.561	0.510
Retired elderly	2.199	1204.000	1201.801
MAiD - projections	0.040	4.993	4.953
Indigenous	0.07	3.776	3.707
Total non-voluntary outsourcing			1273.641

**Table 4.** Voluntary Scenario With MAiD Performed Using Traditional Practice.

Scenarios	Costs ($ CAD billions)	Savings ($ CAD billions)	Cost-benefits ($ CAD billions)
Attempted suicides	3.698	1.025	−2.673
Mentally ill	0.340	1.955	1.615
Homeless	0.178	0.191	0.013
Retired elderly	1.017	54.300	53.284
MAiD - projections	0.408	4.993	4.585
Indigenous	0.583	3.127	2.544
Total voluntary traditional			59.368

**Table 5.** Voluntary Scenario With MAiD Performed Using Outsourcing Approach.

Scenarios	Costs ($ CAD billions)	Savings ($ CAD billions)	Cost-benefits ($ CAD billions)
Attempted suicides	0.361	1.025	0.664
Mentally ill	0.033	1.955	1.922
Homeless	0.017	0.191	0.173
Retired elderly	0.099	54.300	54.201
MAiD - projections	0.040	4.993	4.953
Indigenous	0.06	3.127	3.070
Total voluntary outsourcing			64.983

#### Attempted Suicide

In both voluntary and non-voluntary cases, the traditional approach results in negative cost-benefit (CAD $-2.673 billion), while the out sourcing approach shows a positive economic benefit for the government (CAD $664 million). Unlike for the other cases, for suicides, there is no difference between voluntary and non-voluntary cases for both approaches.

#### Mentally ill

For non-voluntary cases, there is a significant positive cost-benefit difference between the traditional and outsourcing approaches, with the outsourcing approach showing a CAD $10 billion higher benefit (CAD $62.006 vs. CAD $52.107 billion). In voluntary cases, the cost-benefit is much lower in both approaches, though the outsourcing approach still shows a slight improvement (CAD $1.922 vs. CAD $1.615 billion).

#### Homeless

In both non-voluntary and voluntary cases, the outsourcing approach demonstrates a higher benefit than the traditional approach. In non-voluntary cases, the outsourcing approach generates an additional CAD $473 million in benefits, whereas in voluntary cases, it results in an additional CAD $160 million in economic benefits.

#### Retired Elderly

The cost-benefit in non-voluntary cases is significantly higher compared to voluntary cases. The outsourcing approach shows an incremental improvement over the traditional approach in both cases, but the non-voluntary case shows a dramatic positive difference compared to the voluntary case. The outsourcing approach provides an additional CAD $20 billion in benefits (CAD $1201.801 billion compared to CAD $1181.454 billion) in non-voluntary cases, while in voluntary cases, the difference is less with an increase of CAD $918 million (CAD $54.201 billion vs. CAD $53.284 billion).

#### MAiD Projections

The outsourcing approach yields an additional CAD $368 million in both non-voluntary and voluntary cases, with the projected benefits increasing from CAD $4.585 billion (traditional) to CAD $4.953 billion (outsourcing) in both scenarios.

#### Indigenous

The outsourcing approach leads to a substantial additional benefit of CAD $635 million in non-voluntary cases (CAD $3.707 billion vs. CAD $3.072 billion), while in voluntary cases, the increase is CAD $526 million (CAD $3.070 billion vs. CAD $2.544 billion).

While the financial savings from offering MAiD to groups such as those who are suicidal, have mental illness, the homeless, the elderly, current MAiD patients and the indigenous may be substantial, and for a government focused only on economics tempting, the non-economic costs could be far-reaching. One major concern is the potential for societal desensitization to death. If MAiD becomes more widely available, especially to those who are already marginalized, society may begin to view death as an acceptable solution for these populations rather than addressing the underlying issues such as mental health support, poverty, or lack of social services. Furthermore, this could reinforce and perpetuate the marginalization of these groups, leading to an implicit message that their lives are less valuable or less worth saving. For example, offering MAiD to the homeless or individuals with severe mental illnesses could be seen as a shortcut solution to deeper systemic problems, rather than investing in long-term healthcare, education, housing, or social welfare.

The moral dilemma of expanding MAiD to vulnerable populations centers around the ethical trade-off between financial savings and human dignity. While there are clear economic incentives—reduced healthcare costs, fewer long-term care needs, and overall government savings—the fundamental question remains: is it ethical to prioritize cost-saving measures over the sanctity and dignity of human life? Vulnerable populations may be at a higher risk of feeling coerced into choosing MAiD due to financial burdens, social pressure, or a lack of adequate care. Ultimately, the social and ethical costs of expanding MAiD to vulnerable groups may far outweigh the financial benefits, as it risks eroding the value society places on life and further entrenching systemic inequalities.

## Discussion

MAiD is increasingly viewed as a method to cut healthcare costs Medically Assisted Deaths Could Save Millions in Health Care Spending (Malone, 2017; Trachtenberg & Manns, 2017) and it is clear from the results of this study that as several advocates of expanding MAiD have argued, if MAiD is expanded to many marginalized populations within Canada, the government would save money. The results of this analysis show that the savings will be on the order of billions of dollars per year. The savings become even more substantial if killing of Canadian citizens is not extracted from the medical process and instead, carried out by low-skilled government employees or outsourcing is legalized. Other proposed approaches to administering MAiD have also been explored such as the single-serving death chamber in the form of a capsule designed to kill the occupant (Reuters, 2024). To answer the question if automation with the capsule design or other devices would further reduce costs is left for future work.

This entire line of thinking, however, has several ethical weaknesses. Firstly, framing MAiD as a legitimate cost-saving measure devalues the inherent dignity of human life (Aquinas, 1869; Kant & Gregor, 1996; Kass, 2006; Paul, 1995; Schweitzer, 1923), leading to what some refer to as a dangerous societal mindset where death is seen as a convenient and efficient solution to suffering, rather than investing in better care (Nielsen, 2021).

Several ethical frameworks would stand against MAiD as a whole, but especially when it is seen as an economic advantage. For example, the deontological perspective holds that life has intrinsic value, and it is morally wrong to terminate it (Parra Jounou et al., 2024). From the deontological viewpoint, moral actions are guided by principles and duties rather than outcomes. Therefore, ending a life through euthanasia is seen as inherently unethical (Parra Jounou et al., 2024). The deontological approach emphasizes that human life should be respected as an end in itself (Jordan, 2016), and any action to terminate it for financial gain violates the moral duty to preserve life. Similarly, vitalism asserts that human life is inherently valuable and should be preserved at all costs, irrespective of the financial burden of medical treatment or any pain and discomfort that such treatment may cause (Bentham, 1780; Keown, 2018; Mill, 1966).

Allowing individuals to profit from MAiD payments from the government bring up many issues related to conflict of interest and MAiD practioner motivation. Concerns have been expressed about individuals’ motivations for choosing the medical profession, particularly in light of the introduction of MAiD, which may place them in roles that could involve endorsing practices associated with ending life (Nielsen, 2021). First, MAiD as practiced in Canada can be performed by people that are not trained physicians (in fact that is the reason that MAiD proponents claim that it is not called euthanasia in Canada – to be more inclusive to non-doctors that are ending lives (Dobec, 2022)). Requests for MAiD often come from patients whose judgment may be compromised by pain, the side effects of medical treatments, or the influence of clinical depression (Kissane et al., 2001). According to Gallagher et al. “There is evidence of people receiving assisted death when they are structurally vulnerable, and providing death to people for those situations was never intended by the legislation” (Gallagher et al., 2023). Several questions thus arise, such as:

• Several professional organizations, including the World Medical Association, the British Medical Association, the American Medical Association, and the American Psychiatric Association, have also voiced strong opposition to MAiD (Gallagher & Passmore, 2021). Many Canadian physicians struggle with euthanizing their vulnerable patients (Cheng & Wang, 2024). As many physicians opposed participating in euthanasia due to their conscience, ethical beliefs, religious convictions, or adherence to the Hippocratic Oath (Barrett, 2017) and thus will not perform MAiD, there has arisen de facto MAiD specialists who derive a substantive fraction of their income from billable procedures to kill people and thus have a very strong economic incentive to increase MAiD rates (Kelly Grant, 2017; McElroy, 2018). This brings up questions directly related to physician integrity (Sulmasy et al., 2016). Is it ethical for a medical practitioner to profit from killing people?
• What safeguards must be in place to ensure that those that profit from killing people are not influencing patients to select MAiD? For example, physicians and nurses frequently have insufficient time to fully address their patients’ needs (Donnelly, 2017), compounded by significant and growing challenges in both social care and the rising demand for healthcare and social services (Care Quality Commission, 2017). If some individuals have an economic incentive to schedule care in such a way to decrease care for MAiD targets, it raises questions if such pressures influence patient’s requests to MAiD (Keown, 2018). Risks of coercion and exploitation thus apply to MAiD, particularly for vulnerable populations (e.g., those facing poverty or inadequate healthcare) (Gallagher & Passmore, 2021). This trend is already occurring in hospitals, with an increasing emphasis on do-not-resuscitate orders (DNR), the promotion of MAiD, and the acceleration of decisions related to MAiD (University of Wisconsin-Madison, 2022).
• Moreover, institutions have sometimes failed or declined to thoroughly examine questionable practices related to MAiD, which is often coupled with limiting family members’ access to medical records, citing confidentiality or the “best interest” of the deceased (Anderssen, 2023; Coelho et al., 2023). In its 1982 considerations on euthanasia, the Canadian Law Reform Commission noted that the potential for coercion would always be a significant concern (P. S. and P. C. Government of Canada, 2002). How would the system effectively guard against coercion? Does such a safeguard against coercion require recorded surveillance to ensure for example, patients are purposefully made less comfortable (e.g., altering room temperature either too cold or too hot, reduced or eliminated access to drink or tasty food, reduced or eliminated access to alternating pressure mattresses, reducing or withholding pain medication, being given medicine whose side effect is increased discomfort or pain, preventing or delaying visitors to increase isolation and psychological suffering, etc.) What would such surveillance do to patient and medical staff privacy and quality of life?

There have also been concerns raised about the “slippery slope” effect of MAiD (Mildred, 2018; Raikin, 2024; Sulmasy et al., 2016). Nielsen warns “Once we become a MAiD society, MAiD will lose the status as a last-ditch intervention for uncontrollable pain in imminently dying patients. Sick people will begin to see themselves as easily erasable and as excess human specimens. The conviction that it is a wonderful and divine thing to exist will be hollowed out by notions of a life that strives for societal convenience.” (Nielsen, 2021). Habgood expressed concern that the legalization of euthanasia could lead to more far-reaching and potentially more dangerous consequences such as blurring dividing line between active killing and passive letting die as well as potentially leading to the gradual relaxation of regulations and creating pressures on vulnerable individuals, who, due to feelings of uselessness, fear of being a burden, or reluctance to confront the challenges of aging, might be influenced to choose a quicker end to their lives (Habgood, 1974). The safeguards to prevent this from happening cannot be made effective (Gormally, 1995). For instance, in the Netherlands, by the 1990s, more than 50% of euthanasia cases were reported as *non-voluntary* (Remmelink, 1991; van der Maas et al., 1991). This killing of people without their consent has occurred with an almost complete absence of oversight regarding euthanasia practices in the Netherlands (Keown, 2018).

In 1994, a special committee of the Canadian Senate recommended that the law should not permit MAiD, emphasizing concerns about upholding the fundamental social value of respect for human life (S. of Canada, 1994S of Canada, 1994). The New York State Task Force also concluded that allowing physician-assisted suicide would constitute “unwise and dangerous public policy” (New York State Task Force on Life & the Law, 1984). Mann argues that, if legalized, many doctors would adapt their practices and values over time, with healthcare providers potentially incentivized by companies to support MAiD due to its cost-effectiveness (Mann, 1998). This shift could gradually influence the medical profession, with medical students being taught that MAiD is a standard option, leading more doctors to recommend it to patients (Mann, 1998). Her prediction appears to be occurring in Canada now based on the growth of MAiD. Mann also notes that the legalization of PAS could affect families, as once MAiD is no longer illegal, its perceived benefits for busy relatives may become evident and even become viewed as a preferred or commendable option for ending life (Mann, 1998). In this evolving climate, Mann warns that a prolonged, natural death could be seen as unnecessary or extravagant (Mann, 1998). Similarly, Mann argues, attitudes towards death are likely to shift significantly if MAiD is legalized concluding that strong societal expectations could emerge for individuals to choose assisted suicide as soon as their physical abilities decline to the point of being dependent on others in a way that is seen as costly or burdensome (Mann, 1998).

Vulnerable populations, such as the elderly, mentally ill, and homeless, face unique challenges in decision-making processes related to euthanasia, raising significant ethical and practical concerns. These individuals may be particularly susceptible to external pressures, such as economic hardships, lack of adequate healthcare access, or diminished social support systems, which can undermine their ability to make truly autonomous decisions. The introduction of euthanasia—whether voluntary or otherwise—compounds these concerns, as it opens the door to potential exploitation and unintended harm. The distinction between voluntary and non-voluntary euthanasia further complicates moral considerations, particularly when individuals are unable to provide clear consent. Such cases frequently involve individuals who may be experiencing temporary crises or those whose decision-making capacity is compromised. Ethically, this underscores the paramount importance of safeguarding human dignity and preserving the sanctity of life. It is essential that society does not normalize death as a response to complex, systemic challenges, which would risk desensitizing us to the value of life and perpetuating a dangerous precedent that might disproportionately affect the most vulnerable. Thus, any move toward euthanasia must be critically scrutinized to ensure that it does not inadvertently contribute to the marginalization or exploitation of at-risk individuals.

## Alternatives to Killing – Root Cause Elimination

Instead of hiring people to kill marginalized groups, Canada could focus its spending on expanding mental health services, addressing homelessness, improving elderly care, and treating Indigenous people exactly as they treat all other citizens. *The Mental Health Strategy for Canada* programs (Mental Health Commission of Canada, 2012), as well as various provincial initiatives such as *Ontario’s Roadmap to Wellness* and *Quebec’s Action Plan on Mental Health* target expanding mental health services and resources for those suffering from mental illnesses (Government of Ontario, 2024b; Government of Quebec, 2024) . These programs could be broadened to increase access to psychotherapy, counselling, and community-based mental health care. Increased funding for mental health support, psychological services, and comprehensive counselling for patients considering MAiD could reduce the number of MAiD cases by addressing mental health needs early on.

Similarly, homelessness prevention programs could be expanded, emphasizing on affordable housing projects, and support services to address the root causes of homelessness. The *Housing First* national initiative provides immediate access to permanent housing for homeless individuals, without preconditions such as mental health treatment (Housing, 2022b). *Reaching Home* is another program aimed at reducing chronic homelessness by providing funding to community-based organizations for essential services like shelter, food, and healthcare (Housing, 2022a). Scaling up such initiatives could alleviate the conditions that lead to homelessness, addressing a core issue of instability that contributes to poor health outcomes and reducing the demand for MAiD.

For the elderly, the focus could be on expanding access to geriatric care, home support services, and palliative care to address both health and emotional well-being. Canada’s National Dementia Strategy provides a framework for improving the quality of care for seniors living with dementia, focusing on research, early diagnosis, and improving long-term care facilities (P. H. A. of Canada, 2021). Programs such as Ontario’s Home and Community Care Support Services and BC’s Home Health could be expanded to offer more comprehensive care options, including palliative care, mental health support, and caregiver support (Government of Ontario, 2024a; Ministry of Health, BC, 2017).

Canada already invests an enormous amount in indigenous people as a group and this very investment may be undercutting people from becoming self-reliant. Instead, Indigenous people could be treated as any other people in Canada and then based on the government data it would be expected they would thrive as well as, for example, new immigrants from India, which now represent roughly a similar population of the indigenous population – 1.3 million (S. C. Government of Canada, 2024), who have been successful at integrating and obtaining good qualities of life.

Finally, it should be noted, that even in the voluntary scenarios, patients of any group may simply change their minds with appropriate medical care. It has been documented that the desire for a hastened death among terminally ill patients is relatively uncommon, typically temporary, and closely linked to depression and the presence of distressing physical symptoms (Price & Wessely, 2014). Moreover, antidepressant treatment has been found to be effective in managing depression in patients with life-limiting illnesses (Price & Wessely, 2014).

## Conclusions

The study clearly shows that expanding MAiD to include vulnerable populations such as the mentally ill, homeless, elderly, and indigenous groups in Canada could lead to substantial government economic savings. For example, the cost savings associated with the mentally ill could reach up to CAD $63.076 billion, while for the retired elderly population, the savings could be CAD $54.2 billion in the voluntary scenario or as high as CAD $1.2 trillion in a non-voluntary scenario, using traditional methods of providing MAiD in both cases. For the voluntary scenario, the total savings amount to CAD $59 billion for traditional MAiD and CAD $65 billion using an outsourcing approach. Overall, the total savings across all groups in the non-voluntary MAiD scenario are CAD $1.238 trillion (traditional), and CAD $1.273 trillion (outsourcing). The total number of projected deaths in the voluntary MAiD scenario results in 2,674,080 Canadian deaths with a significant portion coming from the mentally ill and elderly groups. This creates a deeply troubling ethical dilemma. Providing such large economic incentives to have the government fund killing its own citizens could easily shift the focus of healthcare policy towards promoting MAiD as a financially attractive option, rather than ensuring that vulnerable individuals receive the care and support they need. Such a system could foster a culture where ending life is seen as preferable to providing adequate care and support, which is dangerous for any society. The government could view MAiD as a cost-saving mechanism, which could lead to policies that implicitly or explicitly encourage individuals to opt for assisted death. The expansion of MAiD in this context risks devaluing human life, particularly among those who are most at risk of societal neglect. This is a dangerous and ethically fraught direction for any policy, as it implicitly encourages a “license to kill” mentality, which is highlighted by the fact the government savings increase substantially using non-medical contract killers. In conclusion, while the potential financial benefits of expanding MAiD are considerable, the ethical costs are far too great. Allowing vulnerable populations to opt for assisted death as a means of saving money for the state undermines the core values of medical ethics and morals. Policymakers must carefully consider these implications before moving forward with any further expansion of MAiD.
